# Antagonistic Feedback Control of Muscle Length Changes for Efficient Involuntary Posture Stabilization

**DOI:** 10.3390/biomimetics9100618

**Published:** 2024-10-11

**Authors:** Masami Iwamoto, Noritoshi Atsumi, Daichi Kato

**Affiliations:** Human Science Research-Domain, Toyota Central R&D Labs., Inc., 41-1, Yokomichi, Nagakute, Aichi 480-1192, Japan; atsumi@mosk.tytlabs.co.jp (N.A.); d-kato@mosk.tytlabs.co.jp (D.K.)

**Keywords:** involuntary posture stabilization, muscle length feedback control, actor-critic reinforcement learning, neutral body posture, human musculosketetal model

## Abstract

Simultaneous and cooperative muscle activation results in involuntary posture stabilization in vertebrates. However, the mechanism through which more muscles than joints contribute to this stabilization remains unclear. We developed a computational human body model with 949 muscle action lines and 22 joints and examined muscle activation patterns for stabilizing right upper or lower extremity motions at a neutral body posture (NBP) under gravity using actor–critic reinforcement learning (ACRL). Two feedback control models (FCM), muscle length change (FCM–ML) and joint angle differences, were applied to ACRL with a normalized Gaussian network (ACRL–NGN) or deep deterministic policy gradient. Our findings indicate that among the six control methods, ACRL–NGN with FCM–ML, utilizing solely antagonistic feedback control of muscle length change without relying on synergy pattern control or categorizing muscles as flexors, extensors, agonists, or synergists, achieved the most efficient involuntary NBP stabilization. This finding suggests that vertebrate muscles are fundamentally controlled without categorization of muscles for targeted joint motion and are involuntarily controlled to achieve the NBP, which is the most comfortable posture under gravity. Thus, ACRL–NGN with FCM–ML is suitable for controlling humanoid muscles and enables the development of a comfortable seat design.

## 1. Introduction

Vertebrates possess numerous muscles despite having relatively few joints. The coordinated activation of these muscles enables involuntary posture stabilization. Muscles exhibit various motion types, such as flexors and extensors, adductors and abductors, and invertors and extortors. For instance, the tibial anterior muscle facilitates both flexion and adduction, complicating the understanding of posture control under gravity.

Neurology and robotics research focuses on how humans control muscles for posture stabilization and intentional movements. Many studies have explored the control of multiple muscles to achieve specific postures or motions. Linear feedback gain control methods, such as proportional–integral–derivative control [1,2] and optimal control algorithms with cost functions [3], estimate muscle activation levels using human musculoskeletal models. These methods predetermine each muscle’s percentage contribution or activation delay for joint motions to realize target postures and movements. However, they struggle to achieve robust motion control in unexpected static or dynamic environments, as muscle contributions and delays can vary with different postures and motions.

Reinforcement learning (RL) has recently emerged as an effective approach for action selection in unknown environments. The actor–critic method, believed to model RL in the basal ganglia [4], has been utilized to estimate muscle activation levels for desired postures or motions in various static and dynamic environments [5,6,7,8,9]. This method simulates involuntary posture stabilization using human musculoskeletal models with anatomical muscle lines [5,8,9] and repeated practice with a mechanistic two–link arm model comprising two joints and six muscles [6,7]. However, learning muscle control policies for posture stabilization under gravity is cost–intensive. Additionally, estimating activation levels of muscles with multiple motion types is challenging, as the method requires the precategorization of muscles into motion types like flexors or extensors.

In our previous study, we developed a musculoskeletal model of an adult male’s right upper limb using MATLAB (MathWorks, Natick, MA, USA) and an actor–critic reinforcement learning (ACRL) system. The actor and critic networks were implemented using a normalized Gaussian network (ACRL–NGN) or deep deterministic policy gradient (DDPG), a widely–used efficient RL algorithm with a deterministic policy, alongside a feedback control model (FCM) of muscle length change (FCM–ML) or joint angle differences (FCM–JA) [10]. Unlike FCM–JA, FCM–ML does not need any precategorization of muscles for motion types. The system efficiently learns involuntary posture stabilization of the right upper extremity under gravity. However, the efficiency and effectiveness of FCM–ML, FCM–JA, ACRL–NGN, and DDPG in achieving desired postures under unexpected static or dynamic environments remain unclear. The feasibility of applying the system to other body parts and involuntary control of multiple muscles for posture under gravity remains obscure as well.

Thus, in the current study, parametric investigations using ACRL–NGN, DDPG, FCM–ML, and FCM–JA were performed for involuntary posture stabilization of the right upper and lower extremities to investigate the learning efficiency and effectiveness of ACRL algorithms and FCMs and discuss how human muscles involuntarily control posture under gravity. Further, we discuss the contribution of the efficient and effective muscle control method for involuntary posture stabilization to the field of clinical or sports science and robotics.

## 2. Materials and Methods

### 2.1. Muscle Control System of the Whole–Body Musculoskeletal Model

**Figure 1:**
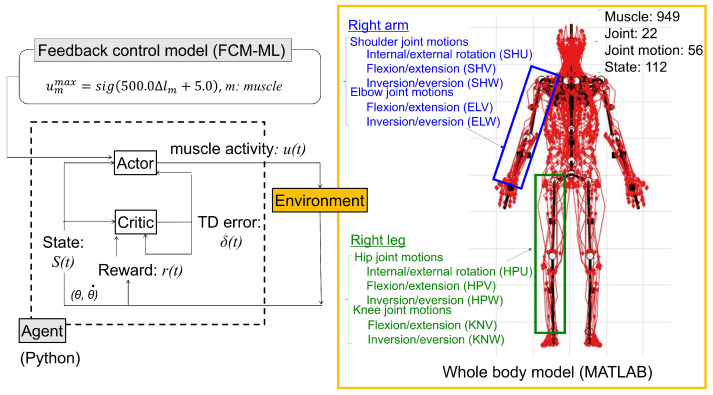
Muscle control system of the whole–body musculoskeletal model based on the actor–critic reinforcement learning (ACRL)–normalized Gaussian network (NGN) and feedback control model (FCM)–muscle length (ML) change algorithms.

Figure 1 illustrates the muscle control system of a whole–body musculoskeletal model employing the ACRL–NGN algorithm with FCM–ML. The model, developed in MATLAB, included 949 muscle lines (excluding the face) and 22 articular joints. In the muscle controller depicted in Figure 1, the state variable s(t) was calculated using the joint angle θ(t) and joint angular velocity $\dot{\theta }\left(t\right)$ at time *t*. Consequently, 112 state variables were defined for 56 joint motions across the entire body. Anthropometric data for the model were sourced from the 3D male anatomy model (Zygote, American Fork, UT, USA). This study examined posture stabilization of five joint motions in the right upper extremity: elbow flexion–extension (ELV) and inversion–eversion (ELW), shoulder internal–external rotation (SHU), flexion–extension (SHV), and inversion–eversion (SHW). It also analyzed five joint motions in the right lower extremity: knee flexion–extension (KNV) and inversion–eversion (KNW), hip internal–external rotation (HPU), flexion–extension (HPV), and inversion–eversion (HPW). According to anatomical texts (e.g., [11]), the initial angle ranges were as follows: ELV (−135° to 17°), ELW (0° to 180°), SHU (−120° to 40°), SHV (−170° to 50°), SHW (−90° to 70°), KNV (−10° to 150°), KNW (−40° to 10°), HPU (−80° to 30°), HPV (−140° to 20°), and HPW (−50° to 40°). The muscle moment arm, determined by the muscle’s line of action relative to joint position, is indicated by muscular force and joint motion. The musculoskeletal model’s biofidelity was validated by comparing predicted muscle moment arms with experimental data from human subjects. Previous studies validated 17 major right upper extremity muscles (Table 1) against experimental data from the literature [10,12,13,14,15,16] and 13 major right lower extremity muscles (Table 1) against data from earlier research [17,18,19,20]. Figure A9 and Figure A10 depict the validation results for the 13 right lower extremity muscles.

In ACRL–NGN, the critic and actor networks were implemented using the NGN and continuous–time formulation of RL [4,21,22]. The state value function V(s(t)) in the critic and action value function $a_{m}\left(s\left(t\right)\right)$ for the *m*th muscle in the actor are represented as follows Equations (1) and (2):

(1) $$V\left(s\left(t\right)\right)=\sum_{i=1}^{K}v_{i}b_{i}\left(s\left(t\right)\right),$$

(2) $$a_{m}\left(s\left(t\right)\right)=\sum_{i=1}^{K}w_{i}^{m}b_{i}\left(s\left(t\right)\right),$$

where $v_{i}$ and $w_{i}^{m}$ are the weights of the critic and actor, respectively; $b_{i}\left(s\left(t\right)\right)$ denotes the base function and is represented by the following Equation (3):

(3) $$\begin{array}{c} b_{i}\left(s\left(t\right)\right)=\frac{B_{i}\left(s\left(t\right)\right)}{\sum_{l=1}^{K}B_{l}\left(s\left(t\right)\right)},\;B_{i}\left(s\left(t\right)\right)=\exp\left[-\sum_{k=1}^{n}{\left(\frac{s_{k}\left(t\right)-c_{k}}{\sigma_{b}^{k}}\right)}^{2}\right], \end{array}$$

where $c_{k}$ denotes the coordinates (dθ, $d\dot{\theta }$) of the center of the activation function, $\sigma_{b}^{k}$, *K*, and *n* represent the constant, the number of base functions, and the number of states s(t), respectively. In this study, K=144,n=112, and $\sigma_{b}^{k}=$ 26.5 and 163.6 for joint angle and joint angular velocity, respectively. The angle difference dθ between the current angle and target angle ranged from $-70^{\circ }$ to $70^{\circ }$, and angular velocity difference $d\dot{\theta }$ between the current angular velocity and target angular velocity ranged from $-300^{\circ }$/s to $300^{\circ }$/s. The target angles of five joint motions were determined using the neutral body posture (NBP) in spaceflight [23], where $\theta_{ELV_{trg}}=-88.0^{\circ },\;\theta_{ELW_{trg}}=0^{\circ },\;\theta_{SHU_{trg}}=-39.0^{\circ },\;\theta_{SHV_{trg}}=-36.0^{\circ },\;\theta_{SHW_{trg}}=36.0^{\circ }$ in the right upper extremity and $\theta_{KNV_{trg}}=43.0^{\circ },\;\theta_{KNW_{trg}}=20.0^{\circ },\;\theta_{HPU_{trg}}=-12.0^{\circ },\;\theta_{HPV_{trg}}=-52.0^{\circ },\;\theta_{HPW_{trg}}=-9.0^{\circ }$ in the right lower extremity. Since the musculoskeletal model had $-30^{\circ }$ of the ELV angle initially, the target angle of ELV was modified from $-58^{\circ }$ (the original angle) to $-88^{\circ }$ to achieve the NBP in this study. The target angular velocities of the five joint motions were set to zero for postural stabilization of the right upper and lower extremities.

The weights of the critic and actors $v_{i}$ and $w_{i}^{m}$ were updated using the following Equations (4) and (5):

(4) $$\begin{array}{ccc} \Delta v_{i} & = & \alpha_{V}\delta \left(t\right)e_{k}\left(t\right), \end{array}$$

(5) $$\begin{array}{ccc} \Delta w_{i}^{m} & = & \alpha_{a}\delta \left(t\right)n_{m}\left(t\right)\exp\left(-0.5V\left(s\left(t\right)\right)\right)\frac{\partial a_{m}\left(s\left(t\right)\right)}{\partial w_{i}^{m}}, \end{array}$$

where $\alpha_{V}$ and $\alpha_{a}$ denote the learning rate of the critic and the actor, respectively. $n_{m}\left(t\right)$ is the white noise function that was randomly determined for each muscle *m* from 0 to 1 at each time step to explore the control output. The symbol δ(t) represents the temporal difference error and is described as follows Equation (6):

(6) $$\begin{array}{ccc} \delta (t) & = & r(s(t))+\gamma V(s(t+1))-V(s(t))\\ & = & r\left(s\left(t\right)\right)+\left(1-\frac{\Delta t}{\tau }\right)V\left(s\left(t+1\right)\right)-V\left(s\left(t\right)\right), \end{array}$$

where γ denotes the discount factor ranging from 0 to 1, and τ denotes a time constant of evaluation; $e_{k}\left(t\right)$ represents the eligibility trace, which is updated using the following Equation (7):

(7) $${\dot{e}}_{k}\left(t\right)=-\frac{1}{\kappa }e_{k}\left(t\right)+\frac{\partial V(s(t))}{\partial w_{k}^{V}},$$

where the symbol κ denotes the time constant of the eligibility trace. In this study, $\alpha_{V}=0.3,$ $\alpha_{a}=0.11$, τ=0.05, and κ=0.05.

The reward function r(s(t)) is defined as follows Equation (8):

(8) $$r\left(s\left(t\right)\right)=\sum_{i=1}^{NJM}\left(\exp\left(-{\left(\frac{d\theta_{i}}{\sigma_{r}}\right)}^{2}\right)+\exp\left(-{\left(\frac{d{\dot{\theta }}_{i}}{\sigma_{r}}\right)}^{2}\right)\right)-c\sum_{m=1}^{N}u_{m}{\left(t\right)}^{2},$$

where NJM(=5), *c*, $\sigma_{r}$, $u_{m}\left(t\right)$, and *N* denote the total number of joint motions, the weight, a constant, muscle activation level of the m-th muscle, and the total number of muscles, respectively. In this study, c=0.01 and $\sigma_{r}=100.0$. The activation level of the m-th muscle $u_{m}\left(t\right)$ is obtained using the following Equation (9):

(9) $$u_{m}\left(t\right)=u_{m}^{max}\mathrm{sig}\left(-A\left(\sum_{k=1}^{K}w_{i}^{m}b_{i}\left(s\left(t\right)\right)+\exp\left(-0.5V\left(s\left(t\right)\right)\right)n_{m}\left(t\right)\right)-B\right),$$

where $u_{m}^{max}$ is the maximum activation level of the m-th muscle, sig() denotes the sigmoid function, *A* and *B* are constants of the sigmoid function, and $n_{m}\left(t\right)$ is the white noise function. In this study, $u_{m}^{max}=1.0$, A=1.0, and B=−4.0.

### 2.2. Simulation Conditions for Learning to Stabilize a Target Posture

We implemented the ACRL–NGN algorithm using Python 3.7 to simulate the posture stabilization of five joint motions of the right upper or lower extremities under gravity. The degrees of freedom, except for these five joint motions, were constrained, and the joint angles calculated using MATLAB (R2013b) were the output, with a time step of 0.01 s. For robust RL in a model–free manner, initial joint angles were randomly selected from specified ranges. In each trial, the motion of the extremity was computed using MATLAB under gravity, with the musculoskeletal model set to the initial angles. The muscle activation level $u_{m}\left(t\right)$ from the actor at time *t* was input to the corresponding muscle, and the joint motions and muscle lengths were calculated using MATLAB. The state s(t), comprising dθ and $d\dot{\theta }$ for each joint motion, was used to calculate the value function V(s(t)) and reward function r(s(t)) to determine the muscle activation level for t+1. Each trial ended at 2.0 s, the simulation’s termination time, and the process was repeated until 300 initial angles were reached.

We used ACRL–NGN or DDPG as the learning algorithm and FCM–ML or FCM–JA as the feedback control model to determine the maximum value of each muscle activation level. We used a DDPG algorithm with the actor–critic method [24], implemented by modifying the Python code of Morvanzhou (https://github.com/MorvanZhou/Reinforcement-learning-with-tensorflow/blob/master/contents/9_Deep_Deterministic_Policy_Gradient_DDPG/DDPG.py, accessed on 8 October 2024). The learning rates of the actor and critic were set to 0.0001, while the τ was set to 0.01. The FCM–ML is described based on the length rate $\Delta l_{m}=\left(l_{m}-l_{m0}\right)/l_{m0}$ of each muscle *m* using Equation (10):

(10) $$u_{m}^{max}=\mathrm{sig}\left(-500.0\cdot \Delta l_{m}+5.0\right),$$

where $l_{m}$ and $l_{m0}$ are the current and equilibrium lengths of each muscle *m*, respectively. In this study, the equilibrium length of each muscle was determined as the length of each muscle when the entire body had an NBP. The FCM–JA is described based on the angle differences $d\theta_{jm}$ in each joint motion jm and the percentage contribution $pc_{m}^{jm}$ of each muscle *m* to each joint motion jm, determined by the following Equation (11):

(11) $$u_{m}^{max}=\mathrm{sig}\left(\sum_{jm=1}^{NJM}pc_{m}^{jm}\cdot d\theta_{jm}\right),$$

where $pc_{m}^{jm}$ takes negative values for flexions of ELV, SHV, and HPV, the extension of KNV, the inversion of ELW, eversions of SHW, HPW, and KNW, and external rotations of SHU and HPU, while positive values were found for the flexion of KNV, extensions of ELV, SHV, and HPV, inversions of SHW, HPW, and KNW, the eversion of ELW, and internal rotations of SHU and HPU. For example, the FCM–JA is described using Equations (A1)–(A20) in Appendix A for the right upper extremity and Equations (A21)–(A49) in Appendix A for the right lower extremity by referring to anatomical texts (e.g., [11]). The absolute values of percentage contribution $pc_{m}^{jm}$ were set to 0.5 and 0.2 for the agonist muscles and synergist muscles, respectively. The value 0.5 was determined based on volunteer test data on muscle strength and muscle activations of the flexors and extensors of the elbow joint during isometric exercise performance, as reported by [25]. The value 0.2 was determined by considering the ratios of activation levels of synergist muscles to those of agonist muscles obtained from experimental test data using electromyography (EMG) [5].

In this study, 12 learning conditions were adopted: two patterns of ACRL–NGN and DDPG for the learning algorithm; three patterns of FCM–ML, FCM–JA, and without any feedback control for the FCM; two patterns of the right upper extremity and right lower extremity for the body part. In each case, the learning calculation was performed for 300 trials to stabilize the target posture, and we compared the simulation results of 12 cases at the 1st, 2nd, 3rd, 20th, and 300th trials (see Table 2). In addition, time–history curves of the joint angles of the ELV, ELW, SHU, SHV, and SHW for the right upper extremity and those of the KNV, KNW, HPU, HPV, and HPW for the right lower extremity were generated.

### 2.3. Simulation Conditions for Arm Motion Predictions under Different Initial Postures

The learning results for the right upper extremity, after 300 trials in the case with ACRL–NGN and FCM–ML, were the most accurate and efficient among all 12 cases. Thus, we performed simulations of postural change from three different initial postures using the muscle activation function obtained after the 300th trial. The muscle activation function corresponds to $w_{i}^{m}$ in Equation (2). We substituted the muscle activation function $w_{i}^{m}$ into Equation (9) and obtained the activation level of the m-th muscle $u_{m}\left(t\right)$ as follows Equations (12)–(14):

(12) $$\begin{array}{ccc} u_{m}^{max} & = & \mathrm{sig}(-500.0\cdot \Delta l_{m}+5.0), \end{array}$$

(13) $$\begin{array}{ccc} \Delta l_{m} & = & \left(l_{m}-l_{m0}\right)/l_{m0}, \end{array}$$

(14) $$\begin{array}{ccc} \sigma (s(t)) & = & \exp(-0.5V(s(t))). \end{array}$$

Equation (12) represents FCM–ML. We performed the posture change simulations from three different initial postures as follows: Case A: the initial posture consisted of $\theta_{ELV}=0^{\circ },\;\theta_{ELW}=0^{\circ },\;\theta_{SHU}=0^{\circ },\;\theta_{SHV}=-90^{\circ }$, and $\theta_{SHW}=0^{\circ }$; Case B: the initial posture consisted of $\theta_{ELV}=0^{\circ },\;\theta_{ELW}=60^{\circ },\;\theta_{SHU}=0^{\circ },\;\theta_{SHV}=40^{\circ }$ and $\theta_{SHW}=0^{\circ }$; Case C: the initial posture consisted of $\theta_{ELV}=-90^{\circ },\theta_{ELW}=0^{\circ },\;\theta_{SHU}=90^{\circ },\;\theta_{SHV}=0^{\circ }$ and $\theta_{SHW}=0^{\circ }$. We determined the posture changes in the whole-body musculoskeletal model, time trajectories of five joint motions in the right upper extremity, and activation levels of major muscles from the simulation results. All the simulations were performed using a Dell OptiPlex5050 computer with an Intel Core i7—6700 processor and 16 GB of DDR4 Memory.

## 3. Results

### 3.1. RL for Stabilization to a Targeted Posture

Table 2 presents the simulation results for posture stabilization of the right upper or lower extremities under gravity. Twelve learning conditions were tested: two algorithms (ACRL–NGN and DDPG), three feedback control models (FCM–ML, FCM–JA, and no FCM), and two body parts (right upper and lower extremities). Learning calculations were performed until the 300th trial for target posture stabilization, with results compared at the 1st, 2nd, 3rd, 20th, and 300th trials. The table numbers indicate the joint angle motions stabilized within a $-5^{\circ }$ to $5^{\circ }$ range of the target angle. For the right upper extremity, FCM–ML with ACRL–NGN stabilized all five joint motions by the 2nd trial and in all subsequent trials, while with DDPG, four joint motions were stabilized by the 3rd and 300th trials. For the right lower extremity, FCM–ML with ACRL–NGN stabilized four joint motions by the 2nd and 300th trials, whereas with DDPG, four joint motions were stabilized by the 300th trial. Cases with FCM–JA and no FCMs achieved stabilization for only one or two joint motions.

**Figure 2:**
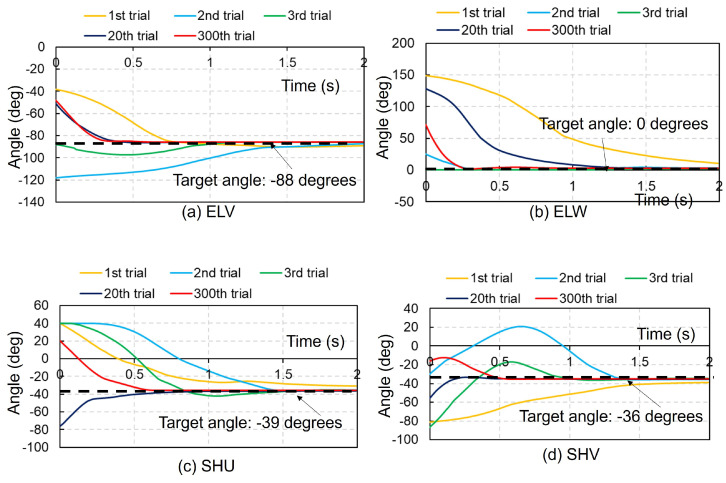

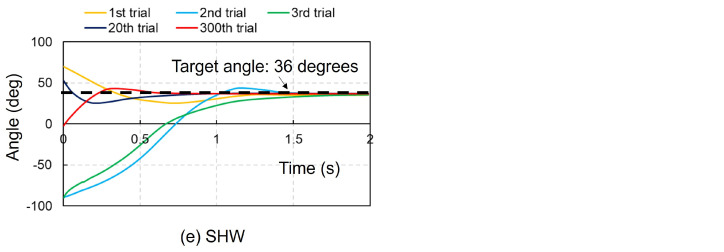
Comparison of the time history of each joint angle in the right upper extremity using the ACRL–NGN and FCM–ML algorithms at the 1st, 2nd, 3rd, 20th, and 300th trials. (**a**) ELV: flexion–extension of the elbow joint, (**b**) ELW: inversion–eversion of the elbow joint, (**c**) SHU: internal–external rotation of the shoulder joint, (**d**) SHV: flexion–extension of the shoulder joint, and (**e**) SHW: inversion–eversion of the shoulder joint. The black dashed lines denote the target angles.

**Figure 3:**
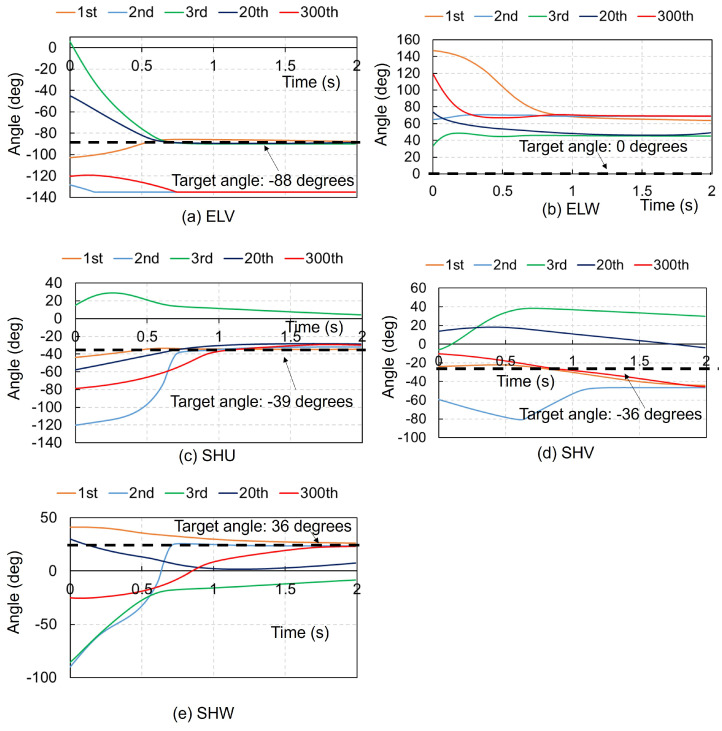
Comparison of the time history of each joint angle in the right upper extremity using the ACRL–NGN and FCM–JA algorithms at the 1st, 2nd, 3rd, 20th, and 300th trials. The black dashed lines denote the target angles.

**Figure 4:**
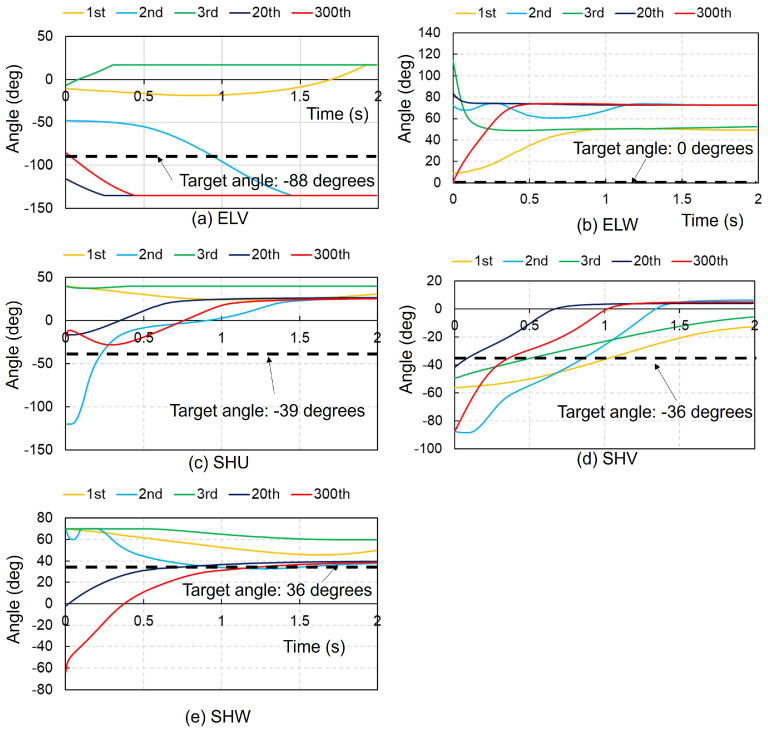
Comparison of the time history of each joint angle in the right upper extremity using the ACRL–NGN algorithm without any FCMs at the 1st, 2nd, 3rd, 20th, and 300th trials. The black dashed lines denote the target angles.

**Figure 5:**
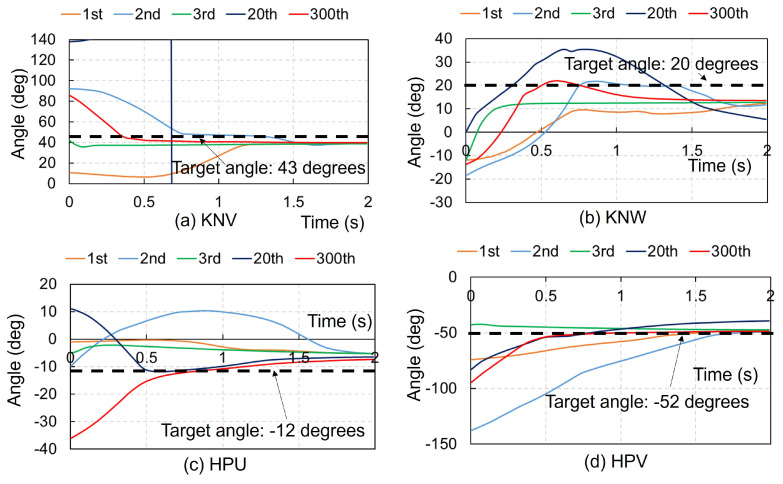

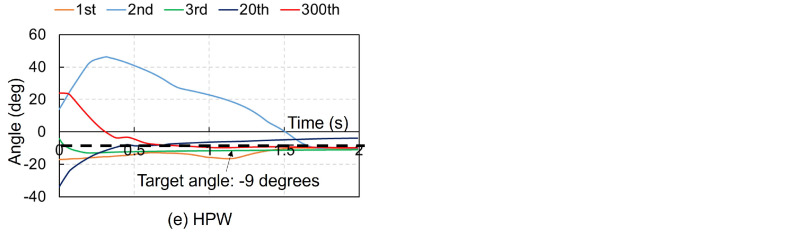
Comparison of the time history of each joint angle in the right lower extremity using the ACRL–NGN and FCM–ML algorithms at the 1st, 2nd, 3rd, 20th, and 300th trials. (**a**) KNV: flexion–extension of the knee joint, (**b**) KNW: inversion–eversion of the knee joint, (**c**) HPU: internal–external rotation of the hip joint, (**d**) HPV: flexion–extension of the hip joint, and (**e**) HPW: inversion–eversion of the hip joint. The black dashed lines denote the target angles.

**Figure A1:**
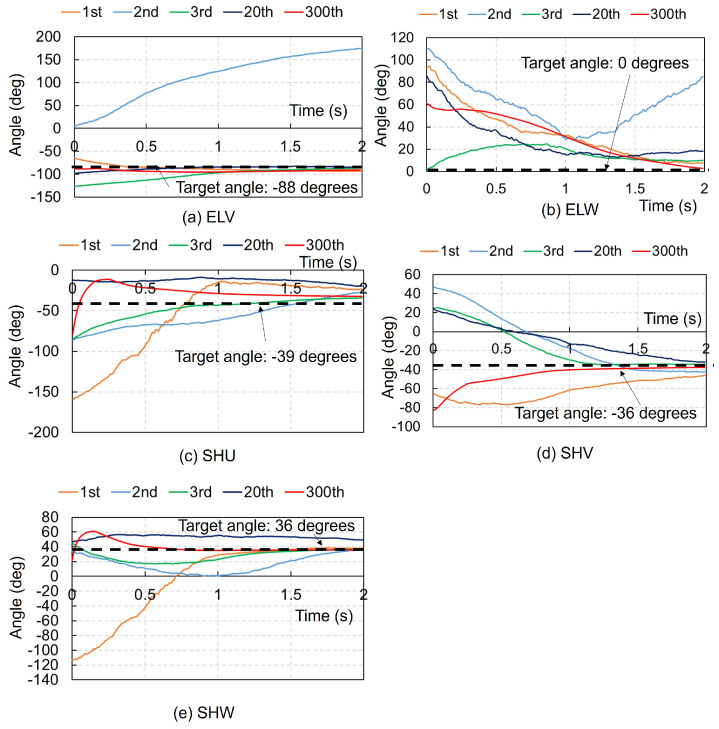
Comparison of the time history of each joint angle in the right upper extremity using the deep deterministic policy gradient (DDPG) and feedback control model (FCM)–muscle length (ML) algorithms at the 1st, 2nd, 3rd, 20th, and 300th trials. (**a**) ELV: flexion–extension of the elbow joint, (**b**) ELW: inversion–eversion of the elbow joint, (**c**) SHU: internal–external rotation of the shoulder joint, (**d**) SHV: flexion–extension of the shoulder joint, and (**e**) SHW: inversion–eversion of the shoulder joint. The black dashed lines denote the target angles.

**Figure A2:**
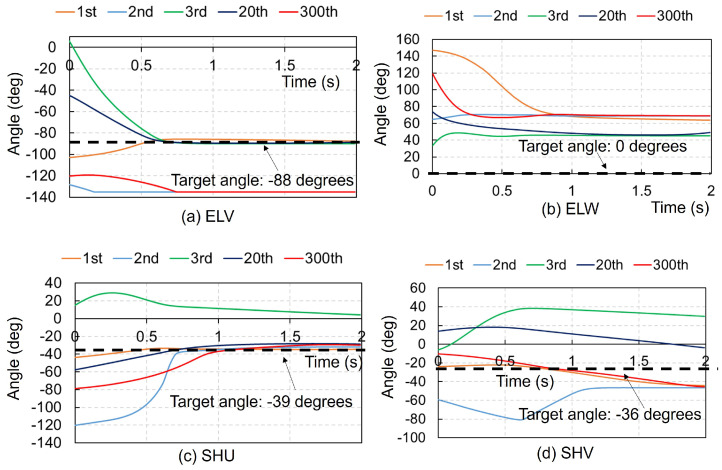

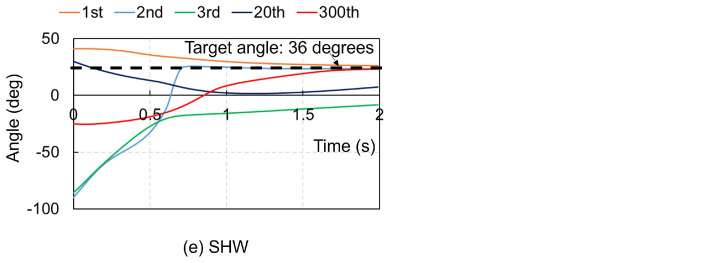
Comparison of the time history of each joint angle in the right upper extremity using the DDPG and FCM–joint angle (JA) algorithms at the 1st, 2nd, 3rd, 20th, and 300th trials. The black dashed lines denote the target angles.

**Figure A3:**
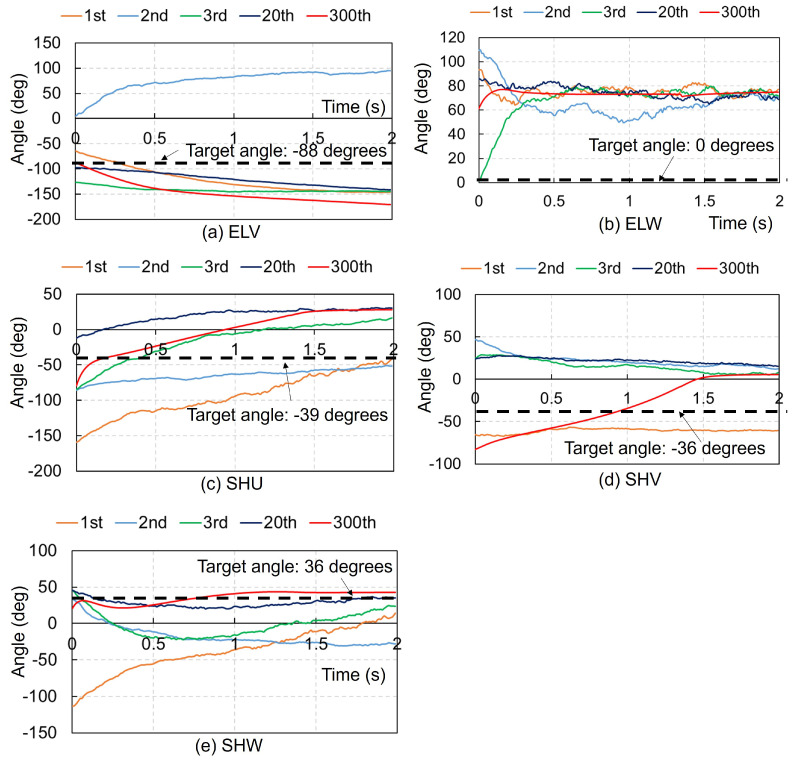
Comparison of the time history of each joint angle in the right upper extremity using the DDPG algorithm without any FCMs at the 1st, 2nd, 3rd, 20th, and 300th trials. The black dashed lines denote the target angles.

**Figure A4:**
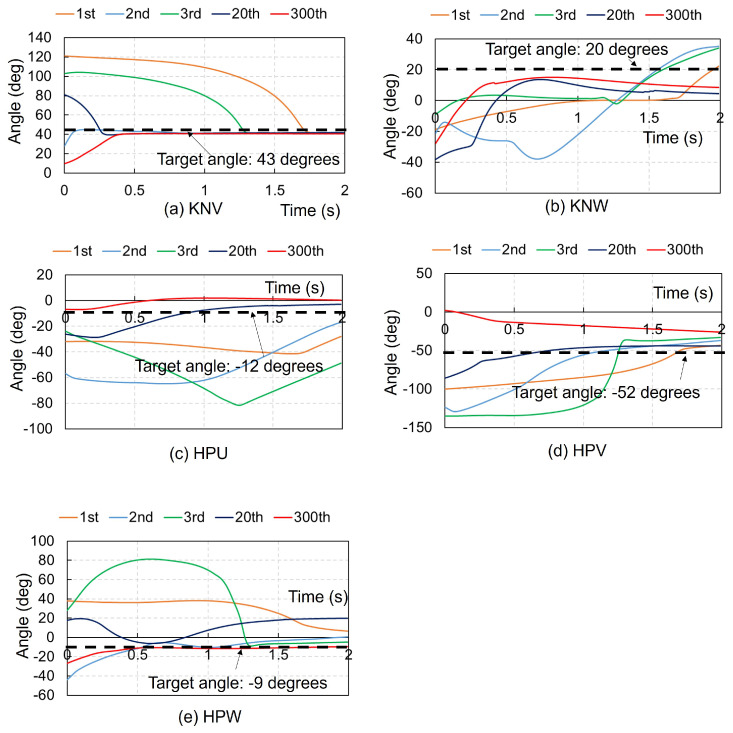
Comparison of the time history of each joint angle in the right lower extremity using the actor–critic reinforcement learning (ACRL)–normalized Gaussian network (NGN) and FCM–JA algorithms at the 1st, 2nd, 3rd, 20th, and 300th trials. (**a**) KNV: flexion–extension of the knee joint, (**b**) KNW: inversion–eversion of the knee joint, (**c**) HPU: internal–external rotation of the hip joint, (**d**) HPV: flexion–extension of the hip joint, and (**e**) HPW: inversion–eversion of the hip joint. The black dashed lines denote the target angles.

**Figure A5:**
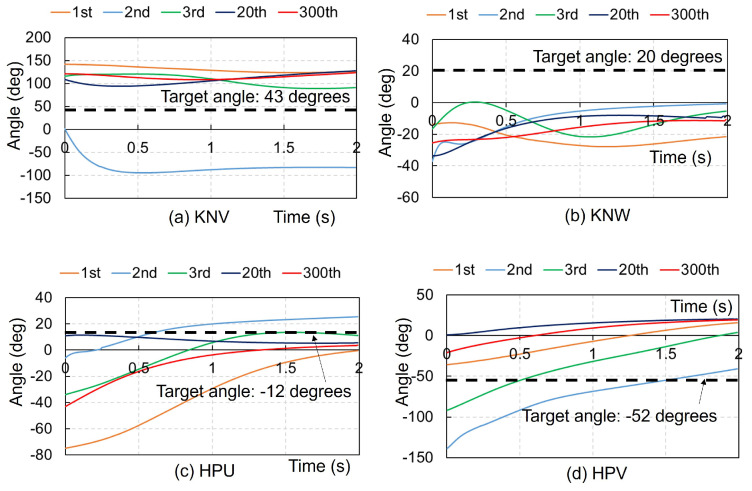

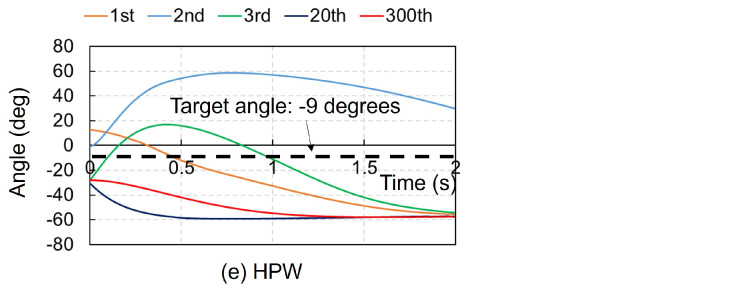
Comparison of the time history of each joint angle in the right lower extremity using the ACRL–NGN algorithm without any FCMs at the 1st, 2nd, 3rd, 20th, and 300th trials. The black dashed lines denote the target angles.

**Figure A6:**
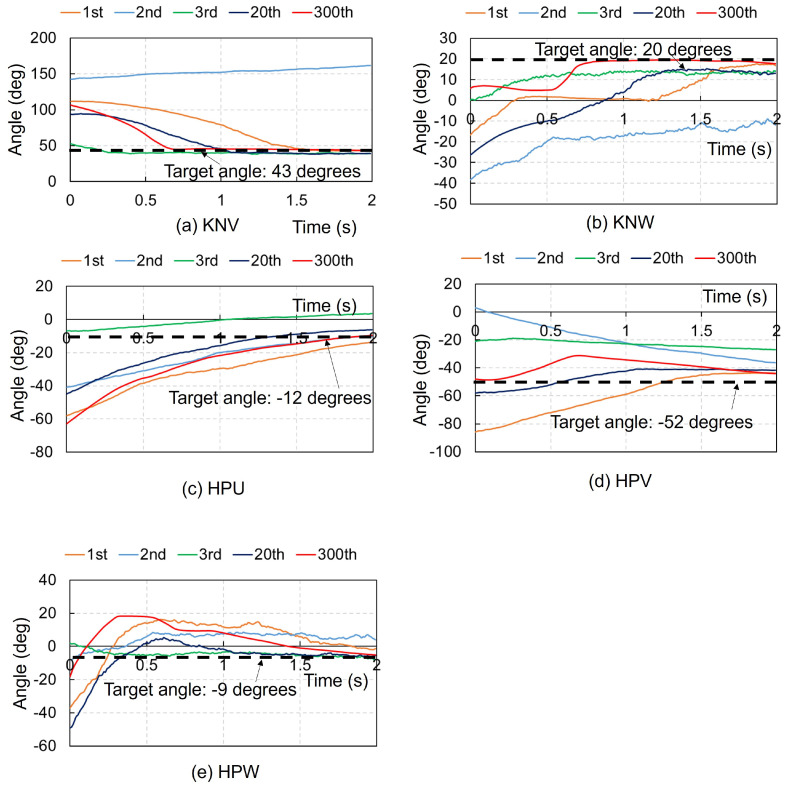
Comparison of the time history of each joint angle in the right lower extremity using the DDPG and FCM–ML algorithms at the 1st, 2nd, 3rd, 20th, and 300th trials. The black dashed lines denote the target angles.

**Figure A7:**
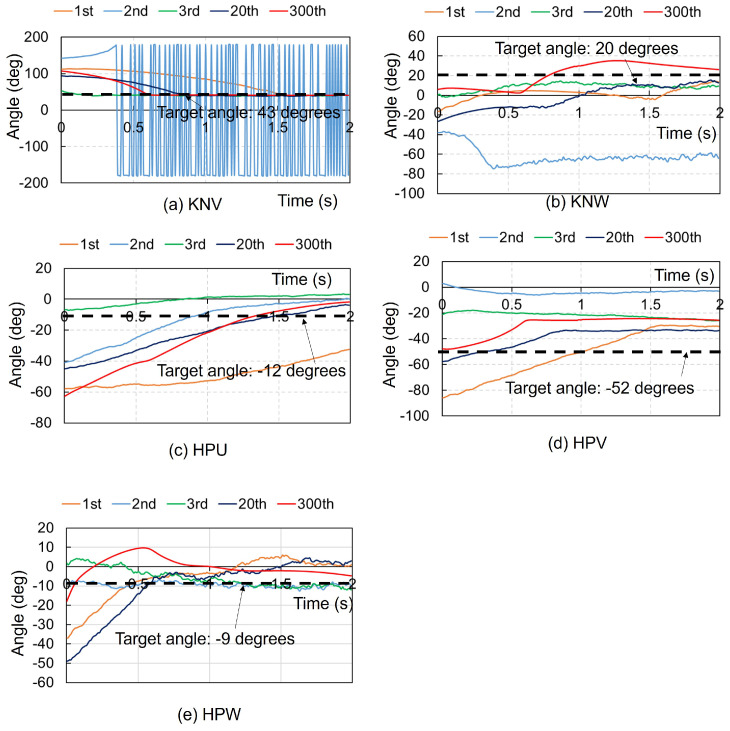
Comparison of the time history of each joint angle in the right lower extremity using the DDPG and FCM–JA algorithms at the 1st, 2nd, 3rd, 20th, and 300th trials. The black dashed lines denote the target angles.

**Figure A8:**
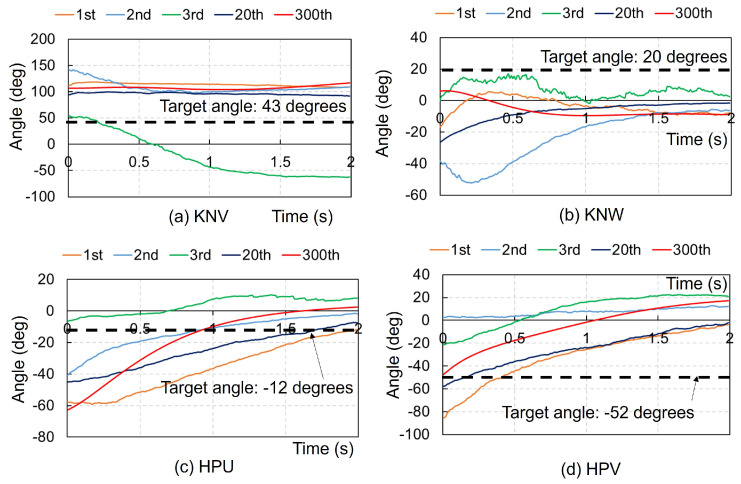

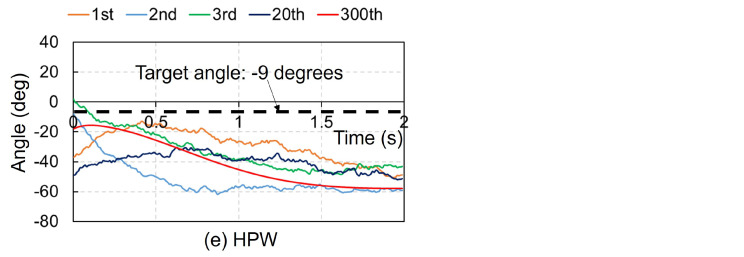
Comparison of the time history of each joint angle in the right lower extremity using the DDPG algorithm without any FCMs at the 1st, 2nd, 3rd, 20th, and 300th trials. The black dashed lines denote the target angles.

Figure 2 presents the time history of each joint angle in the right upper extremity using the ACRL–NGN and FCM–ML algorithms at the 1st, 2nd, 3rd, 20th, and 300th trials. Despite the random initial angles, each joint stabilized at the target angle by 2 s. Both algorithms achieved the target posture (NBP) for all five joint motions by the 2nd trial, remaining stable until the 300th trial. Figure 3 shows the time history using the ACRL–NGN and FCM–JA algorithms over the same trials. Here, the target posture was reached only for the ELV and SHU in the 1st trial and solely for the ELV in the 3rd and 20th trials. Figure 4 illustrates the time histories using the ACRL–NGN algorithm without any FCMs, showing that the target posture was achieved only for SHW in the 2nd, 20th, and 300th trials. Figure 5 compares the time history of each joint angle in the right lower extremity using the ACRL–NGN and FCM–ML algorithms across the trials, displaying that for all four joint motions, except KNW, the posture of the right upper extremity reached the target posture by the 2nd and 300th trials. Figure A1, Figure A2, Figure A3, Figure A4, Figure A5, Figure A6, Figure A7 and Figure A8 show the results of comparisons for additional cases.

### 3.2. Arm Motion Predictions from Different Initial Postures

**Figure 6:**
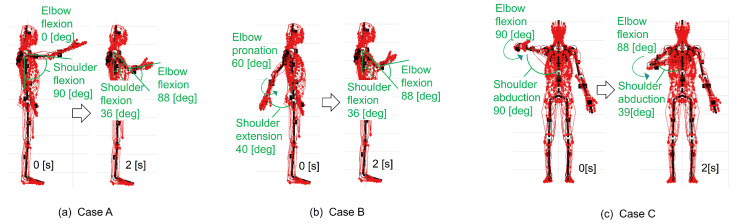
Simulation result of posture change during 2 s of the right upper extremity, obtained using muscle control functions obtained at the 300th trial with the ACRL–NGN and FCM–ML algorithms. (**a**) Case A: an initial posture consisting of ELV: $0^{\circ }$, ELW: $0^{\circ }$, SHU: $0^{\circ }$, SHV: $-90^{\circ }$, and SHW: $0^{\circ }$; (**b**) Case B: an initial posture consisting of ELV: $0^{\circ }$, ELW: $60^{\circ }$, SHU: $0^{\circ }$, SHV: $40^{\circ }$, and SHW: $0^{\circ }$; (**c**) Case C: an initial posture consisting of ELV: $-90^{\circ }$, ELW: $0^{\circ }$, SHU: $90^{\circ }$, SHV: $0^{\circ }$, and SHW: $0^{\circ }$.

Figure 6 illustrates the right upper extremity posture changes over 2 s, predicted from three initial postures using muscle control functions at the 300th trial with ACRL–NGN and FCM–ML algorithms. Figure 6a presents a side view for Case A, starting with shoulder forward flexion at $-90^{\circ }$ and elbow extension at $0^{\circ }$, achieving the target posture (NBP) at 2 s. Figure 6b shows a side view for Case B, with an initial backward shoulder extension of $40^{\circ }$ and elbow inversion of $60^{\circ }$, also reaching the target posture. Figure 6c offers a front view for Case C, beginning with shoulder external rotation at $90^{\circ }$ and elbow flexion at $-90^{\circ }$, successfully attaining the target posture.

**Figure 7:**
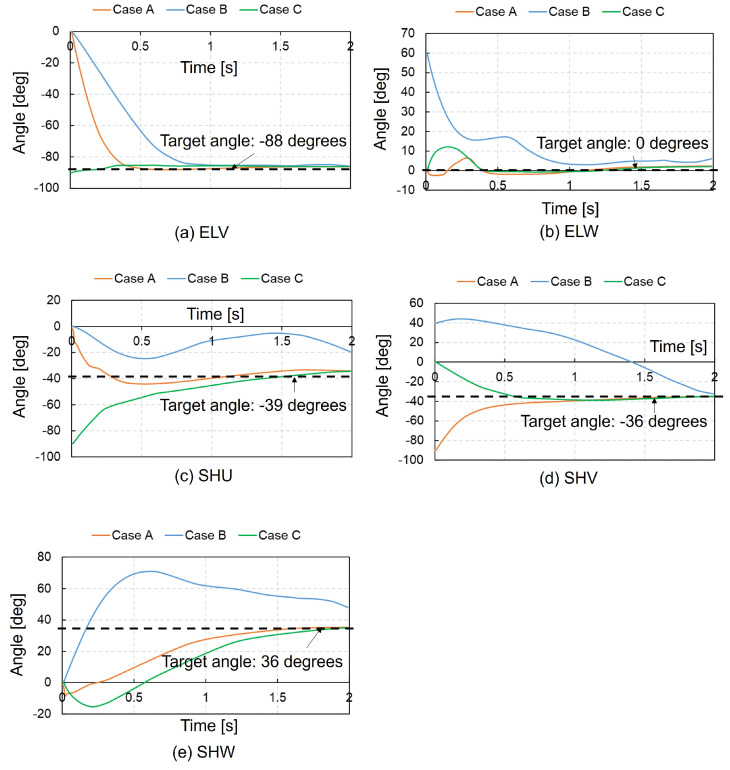
Comparison of each joint angle time history from posture change simulation results during 2 s of the right upper extremity using muscle control functions obtained at the 300th trial with the ACRL–NGN and FCM–ML algorithms. Case A: an initial posture consisting of ELV: $0^{\circ }$, ELW: $0^{\circ }$, SHU: $0^{\circ }$, SHV: $-90^{\circ }$, and SHW: $0^{\circ }$; Case B: an initial posture consisting of ELV: $0^{\circ }$, ELW: $60^{\circ }$, SHU: $0^{\circ }$, SHV: $40^{\circ }$, and SHW: $0^{\circ }$; Case C: an initial posture consisting of ELV: $-90^{\circ }$, ELW: $0^{\circ }$, SHU: $90^{\circ }$, SHV: $0^{\circ }$, and SHW: $0^{\circ }$. The black dashed lines denote the target angles.

**Figure 8:**
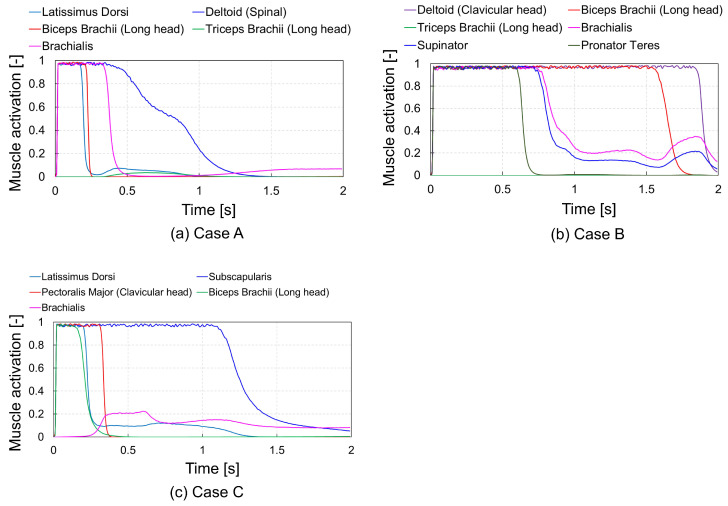
Comparison of each muscle activation time history from posture change simulation results during 2 s of the right upper extremity using muscle control functions obtained at the 300th trial with the ACRL–NGN and FCM–ML algorithms. (**a**) Case A: an initial posture consisting of ELV: $0^{\circ }$, ELW: $0^{\circ }$, SHU: $0^{\circ }$, SHV: $-90^{\circ }$, and SHW: $0^{\circ }$; (**b**) Case B: an initial posture consisting of ELV: $0^{\circ }$, ELW: $60^{\circ }$, SHU: $0^{\circ }$, SHV: $40^{\circ }$, and SHW: $0^{\circ }$; (**c**) Case C: an initial posture consisting of ELV: $-90^{\circ }$, ELW: $0^{\circ }$, SHU: $90^{\circ }$, SHV: $0^{\circ }$, and SHW: $0^{\circ }$.

Figure 7 presents the joint angle time histories obtained from three simulations. In Cases A and C, the five joint angles closely match the target angles. However, in Case B, the SHU and SHW shoulder joint angles deviate by $20^{\circ }$ from their targets, while the ELV, ELW, and SHV angles remain aligned with the targets. Figure 8 illustrates the activation levels of major muscles during posture change simulations. In Case A, the latissimus dorsi and deltoid spinal activities, responsible for shoulder extension, initially increase, while the biceps brachii long head and brachialis, responsible for elbow flexion, are only slightly activated to achieve the target posture. In Case B, the deltoid clavicular head and biceps brachii long head, which flex the shoulder and elbow joints, respectively, initially show increased activity; in contrast, the triceps brachii long head, which extends the elbow joint, is slightly activated. The deltoid, clavicular head and biceps brachii long head sustained their activation for over 1.5 s. Additionally, the supinator and pronator teres, responsible for supinating and pronating the elbow joint, initially show increased activities, which are sustained for over 2 s. The target posture is achieved with the aid of these muscle activities. In Case C, the pectoralis major clavicular head and subscapularis, which internally rotate the shoulder joint, initially show increased activity, with the subscapularis remaining active until 0.3 s after the simulation onset. The activity of the biceps brachii long head, which flexes the elbow joint, increases until 0.3 s after simulation onset and then decreases, whereas the brachialis maintains its activation level between 0.1 and 0.2 until 2 s, thus achieving the target posture.

## 4. Discussion

### 4.1. Comparisons between FCM–ML and FCM–JA

The results obtained in this study indicate that the ACRL–NGN algorithm combined with FCM–ML is the most effective for achieving desired postures in both the right upper and lower extremities, followed by the DDPG algorithm with FCM–ML (see Table 2 and Figure 2). This suggests that muscle control, informed by feedback on muscle length changes, is crucial for determining maximum muscle activation levels and ensuring efficient postural control. Muscle spindles, which are sensory receptors in nearly all muscles, relay information about muscle length changes and stretching speed to the central nervous system (CNS). The CNS uses these data to calculate the position and movement of the extremities, which are essential for motor control, posture maintenance, and stable gait [26,27]. In FCM–ML, the maximum activation level of the m-th muscle is uniquely determined by the muscle length change $\Delta l_{m}$ in Equation (13). An increase in $\Delta l_{m}$ enhances the muscle activation level and the muscle force, promptly achieving the target posture. Moreover, if the current joint angle is more flexed than the target angle, the $\Delta l_{m}$ and muscular forces of extensors become larger than those of flexors, thereby extending the joint and vice versa. FCM–ML retains antagonistic feedback control of muscle length change $\Delta l_{m}$.

**Figure A9:**
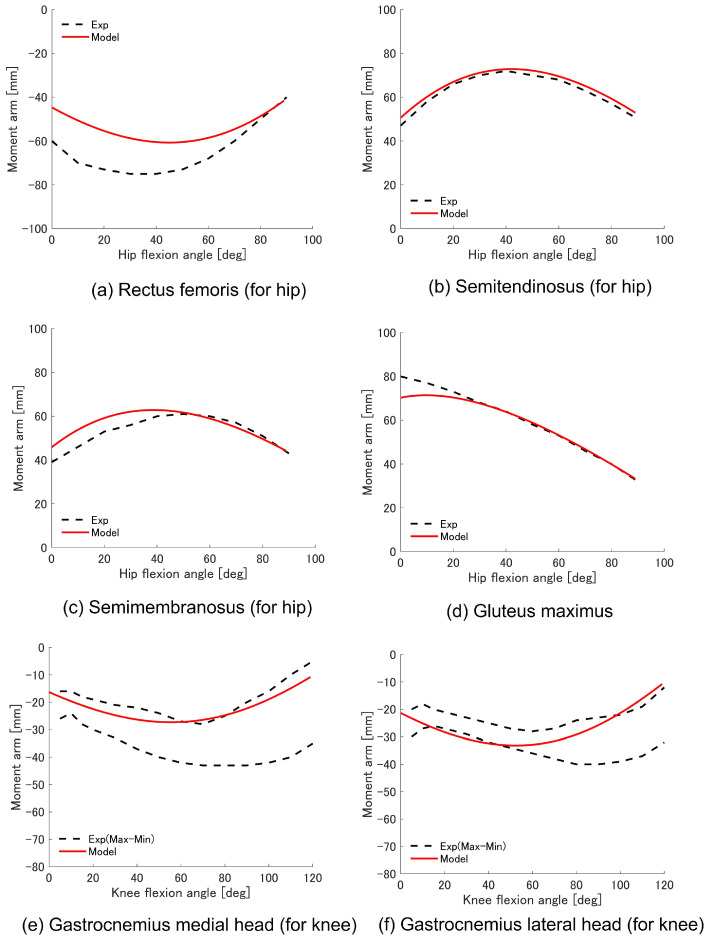

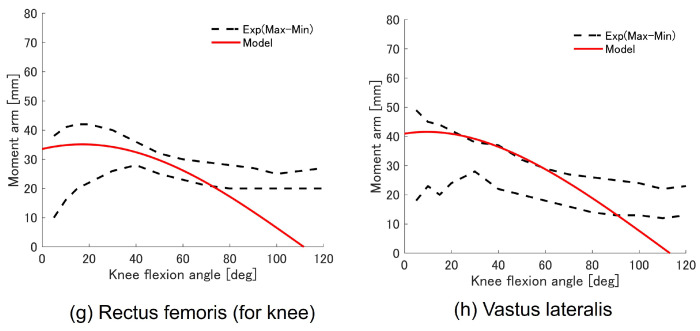
Comparison of moment arm versus knee or hip flexion angles between model prediction and test data.

**Figure A10:**
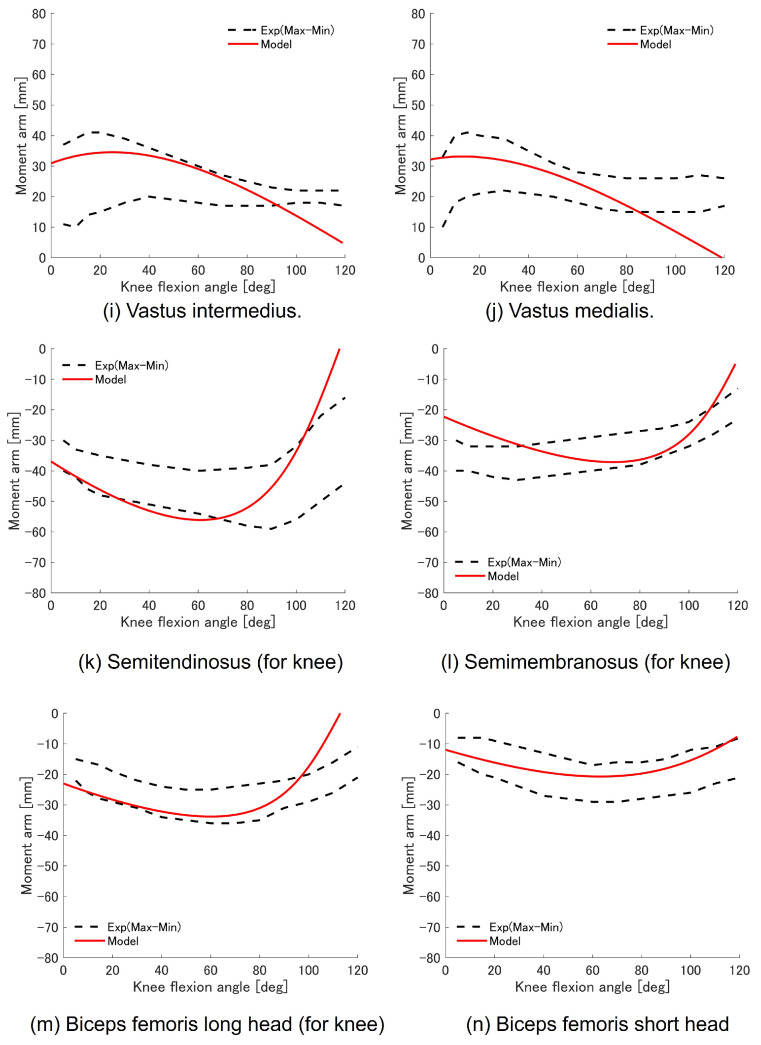

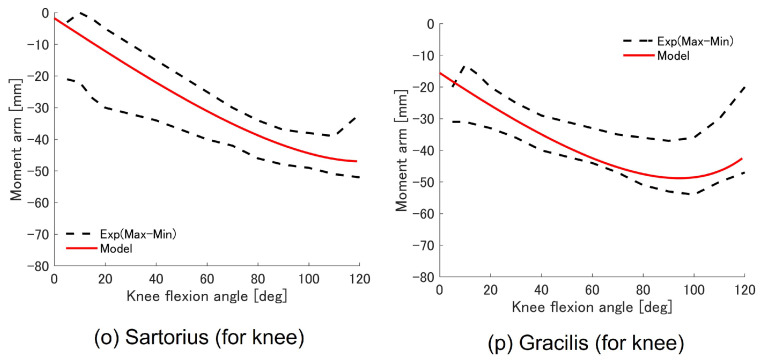
Comparison of moment arm versus knee or hip flexion angles between model prediction and test data.

Muscle forces are highly dependent on fiber length. When muscle–tendon compliance is low (i.e., a small ratio of tendon slack length to optimal muscle fiber length), muscle fiber operating length (reflected by muscle length change $\Delta l_{m}$) is primarily influenced by joint angles and muscle moment arms. Conversely, when compliance is high, it depends more on activation level and force–length–velocity effects [28]. Since the current model excluded tendon elements and did not simulate high muscle–tendon compliance in upper or lower extremity motions, joint angles and muscle moment arms predominantly determine the muscle length change $\Delta l_{m}$. Each muscle in our musculoskeletal model has a moment arm comparable to the human body (refer to [10] for upper extremity muscles and Figure A9 and Figure A10 for lower extremity muscles). Thus, joint angles are assumed to determine the muscle length change $\Delta l_{m}$ and muscle force. However, FCM–JA cases stabilized posture for only one or two joint motions (see Table 2). In FCM–JA, the maximum activation level of the m-th muscle is derived from joint angles (see Equation (11)) but not uniquely determined, as the percentage contribution $pc_{m}^{jm}$ in Equation (11) varies with joint motion types (flexion–extension, inversion–eversion, and internal–external rotations), muscle roles (agonist or synergist for each joint motion) (see Equations (A1)–(A20) for the right upper extremity and Equations (A21)–(A49) for the right lower extremity in Appendix A), and muscle activation delay. The percentage contribution must be predetermined using the anatomical references (e.g., [11]) and EMG experimental data. While it can be predetermined for posture stabilization in expected static or dynamic environments and intentional, voluntary motion, as joint motion type, muscle role, and activation delay are known (see [1,2,5,8,9]), it cannot be predetermined for involuntary posture stabilization in unexpected static or dynamic environments. This phenomenon elucidates why the cases employing FCM–JA demonstrated minimal posture stabilization for the right upper and lower extremities.

### 4.2. Comparisons between ACRL–NGN and DDPG

DDPG, a common RL algorithm for continuous control, learns a deterministic policy via the actor–critic method. However, it suffers from severe overestimation bias due to using a single critic for function approximation, as the actor–network is trained to execute actions with the highest value estimate [29,30]. To address this, the twin–delayed deep deterministic policy gradient (TD3) was proposed, using the minimum value from double critic networks for value estimation with exploration noise [29]. We employed the DDPG and ACRL–NGN algorithms to learn to move extremities from various initial postures to target postures. In our simulations with FCM–ML, while DDPG achieved the target posture in four joint angle motions, the ACRL–NGN algorithm outperformed DDPG in efficiency and accuracy (see Table 2). This likely stems from DDPG’s overestimation issue. Conversely, ACRL–NGN incorporates a continuous state space using a Gaussian softmax network and a noise–induced muscle activation function [10], enhancing performance akin to exploration noise in TD3.

### 4.3. Comparisons with Previous Iterative Learning Methods

Previous studies identified muscle synergies as groups of co-activated muscles to reduce redundancy in the human musculoskeletal system and developed an iterative learning controller to coordinate these synergies for complex tasks [31,32,33]. However, about 40 iterations are required to achieve the target motion, and synergy patterns must be adjusted for specific tasks. In contrast, our method does not necessitate muscle categorization for tuning synergy patterns and achieves target postures or motions with significantly fewer iterations (see Table 2). FCM–JA is a muscle synergy control method. For the upper extremity, the long, lateral, and medial heads of triceps brachii form one group, while the brachialis and brachioradialis form another, as indicated in Equations (A1)–(A20). In each reinforcement learning trial, the initial angles of SHV, SHW, ELV, and ELW vary randomly within the ranges of −170° to 50°, −90° to 70°, −135° to 17°, and 0° to 180°, respectively. Consequently, the upper extremity simulated in this study exhibits various joint motions except ELV. The $pc_{m}^{jm}$ contributions of 0.5 and 0.2 for the agonist and synergist muscles in Equation (11) are valid for ELV but not for the other joint motions. Similar to the results obtained in previous synergy control studies, our findings indicate that FCM–JA requires tuning of synergy patterns for specific joint motions, unlike FCM–ML.

### 4.4. Versatility of ACRL–NGN with FCM–ML to Other Body Parts

In stabilizing the target posture for the right lower extremity, the ACRL–NGN and FCM–ML algorithms achieved target angles for only four joint motions, compared to five joint motions in the right upper extremity. This discrepancy likely arises because the lower extremity contains more biarticular muscles than the upper extremity: nine in the lower extremity (tensor fasciae latae, rectus femoris, gracilis, sartorius, biceps femoris long head, semitendinosus, semimembranosus, gastrocnemius, and plantaris) and three in the upper extremity (triceps brachii long head and bicep brachii long and short heads). Muscle length, influenced by two joints, is severely affected by the tendons in biarticular muscles [28]. The current model omits tendon elements, rendering muscle length feedback ineffective for the lower extremities. Future studies should incorporate a muscle–tendon complex model.

Vertebrates, excluding humans, like dogs and horses, possess different joint motion ranges. Understanding their muscle control mechanisms requires specific data on muscular motion types (e.g., flexors or extensors), muscle roles (e.g., agonist or synergist), and activation patterns from experimental EMG data alongside anthropometric data. However, the feedback method of muscle length change (FCM–ML), combined with musculoskeletal anthropometric data, aids in comprehending their muscle control mechanisms because it does not require specific data on such categorization of muscles. This result implies that vertebrate muscles are fundamentally controlled without classifying flexors or extensors or distinguishing between agonists and synergists necessary to explore muscular synergy patterns for targeted joint motion.

### 4.5. Application Prospects of ACRL–NGN with FCM–ML

ACRL–NGN with FCM–ML does not require muscle categorization for tuning synergy patterns and thus achieves targeted postures or motions with fewer iterations compared to the existing methods (Section 4.3). Therefore, this method is suitable for activating human–like robots with multiple muscle lines, such as a humanoid McKibben arm with multifilament artificial muscles [34]. Additionally, the simulation results presented in Figure 6, Figure 7 and Figure 8 highlight the muscles that should be trained to efficiently achieve targeted motions and thus benefit athletes and patients undergoing rehabilitation. According to the force–length curve of the Hill–type muscle model, each muscle generates maximal force at its natural length [35]. The results of this study indicate that the maximum muscle activation level depends on the difference Δl between the current and target posture muscle lengths (Equation (12)). The NBP is used as the target posture, as each muscle length in the NBP is considered to be the natural length. A large Δl promotes activation via the sigmoid function described in Equation (12), whereas a small Δl inhibits activation but results in a stronger contractile force, allowing the muscle’s current length to approach the target length, and thus, the NBP. Another target posture with an ELV angle of $-30^{\circ }$ was set while maintaining the other joint motions in their original states, and learning calculations using ACRL–NGN and FCM–ML were performed until the 300th trial. The ELV stabilized at $-88^{\circ }$, similar to that observed in the NBP, by the 300th trial, demonstrating that feedback control of muscle–length changes efficiently stabilizes the NBP under gravity without voluntary muscle controls. This result suggests that the NBP is the most comfortable posture for humans [36,37,38], underscoring the potential of ACRL–NGN with FCM–ML in comfortable seat and chair design.

### 4.6. Study Limitations

Nevertheless, the results obtained in this study are influenced by several limitations. First, we did not incorporate tendon elements into the analysis. Second, the time history curves of the muscle activation levels predicted during the three posture change simulations were not validated against EMG data due to the lack of experimental data on arm motions obtained using adult male participants. Further investigations are necessary to replace the current muscle model with a muscle–tendon complex model that incorporates a Hill–type muscle model with serial damping and eccentric force–velocity, similar to the one proposed by Haeufle et al. [39]. Such a modified model is expected to reveal the effects of tendon elements and biarticular muscles on posture stabilization and various motions (e.g., gait and jumping) of the extremities. Additionally, volunteer tests should be conducted to acquire EMG data of surface muscles in the extremities, as described in our previous studies [5,8], and to examine the validity of the predicted muscle activation levels to enhance the biofidelity of the proposed human musculoskeletal model and muscle controller.

## 5. Conclusions

ACRL–NGN, which reflects basal ganglia activity with FCM–ML simulating muscle spindle feedback, achieved the most efficient involuntary posture stabilization among all control methods tested. This approach does not require muscular synergy pattern control classifying flexors or extensors or distinguishing agonists from synergists. Instead, it relies on antagonistic feedback control based on changes in muscle length; thus, this method, which indicates muscles that should be trained for efficiently achieving targeted motions, can be applied to all vertebrate muscles, humanoids with multiple muscles, and in rehabilitation of athletes or patients. Additionally, this method effectively achieves involuntary NBP stabilization, i.e., the most comfortable posture for humans under gravity. This result implies that human body muscles may involuntarily control posture to attain the NBP under gravity, suggesting the potential applicability of the proposed method to comfortable seat design. In future studies, we will replace the current muscle model with a muscle–tendon complex model and apply the ACRL–NGN and FCM–ML algorithms to intentional movements.

## Figures and Tables

**Table 1:** Muscles for validation of their momemt arms.

Upper Extremity	Lower Extremity
Deltoid anterior	Rectus femoris
Deltoid middle	Semitendinosus
Deltoid posterior	Semimembranosus
Teres major	Biceps femoris (long head)
Teres minor	Biceps femoris (short head)
Supraspinatus	Gluteus maximus
Infraspinatus	Gastrocnemius (medial head)
Subscapularis	Gastrocnemius (lateral head)
Biceps brachii (long head)	Vastus lateralis
Biceps brachii (short head)	Vastus intermedius
Triceps brachii (long head )	Vastus medialis
Triceps brachii (lateral head)	Sartorius
Triceps brachii (medial head)	Gracilis
Brachialis	
Brachioradialis	
Pronator teres	
Anconeus	

**Table 2:** Simulation results of posture stabilization of the upper extremity or lower extremity under gravity.

	Learning Algorithm	FCM	1st Trial	2nd Trial	3rd Trial	20th Trial	300th Trial
Right upper extremity	ACRL	Muscle length (ML)	3	5	5	5	5
		Joint angle (JA)	1	0	1	1	0
		None	0	1	0	1	1
	DDPG	Muscle length (ML)	1	1	4	2	4
		Joint angle (JA)	1	0	1	0	1
		None	1	0	0	1	0
Right lower extremity	ACRL	Muscle length (ML)	3	4	3	0	4
		Joint angle (JA)	2	2	1	1	2
		None	0	0	0	0	0
	DDPG	Muscle length (ML)	3	1	1	0	4
		Joint angle (JA)	1	1	2	1	2
		None	1	0	0	0	0

## Data Availability

The original data and code presented in the study are openly available for anonymous peer review at https://osf.io/sc3zt/?view_only=6c9b73083bcc42a380f8383f1978d21f, accessed on 8 October 2024. AI or AI-assisted tools were not used in drafting any aspect of this manuscript.

## Appendix A

The FCM–JA is described based on the angle differences of the five joint motions using the following Equations (A1)–(A20) for the right upper extremity.

(A1) $$u_{1:Deltoid\;anterior}^{max}=sig\left(-0.5d\theta_{SHV}+0.5d\theta_{SHW}\right),$$

(A2) $$u_{2:Deltoid\;middle}^{max}=sig\left(-0.5d\theta_{SHU}\right),$$

(A3) $$u_{3:Deltoid\;posterior}^{max}=sig\left(0.2d\theta_{SHV}-0.2d\theta_{SHW}\right),$$

(A4) $$u_{4:Teres\;major}^{max}=sig\left(0.5d\theta_{SHU}+0.5d\theta_{SHW}\right),$$

(A5) $$u_{5:Teres\;major}^{max}=sig\left(-0.5d\theta_{SHW}\right),$$

(A6) $$u_{6:Supraspinatus}^{max}=sig\left(-0.2d\theta_{SHU}\right),$$

(A7) $$u_{7:Infraspinatus}^{max}=sig\left(-0.5d\theta_{SHW}\right),$$

(A8) $$u_{8:Subscapularis}^{max}=sig\left(0.5d\theta_{SHU}+0.5d\theta_{SHW}\right),$$

(A9) $$u_{9:Coraco\;brachialis}^{max}=sig\left(-0.2d\theta_{SHV}+0.2d\theta_{SHW}\right),$$

(A10) $$u_{10:Biceps\;brachii\;long}^{max}=sig\left(-0.5d\theta_{SHV}-0.2d\theta_{SHW}-0.5d\theta_{ELV}+0.2d\theta_{ELW}\right),$$

(A11) $$u_{11:Biceps\;brachii\;short}^{max}=sig\left(-0.5d\theta_{SHV}-0.2d\theta_{SHW}-0.5d\theta_{ELV}+0.2d\theta_{ELW}\right),$$

(A12) $$u_{12:Triceps\;brachii\;long}^{max}=sig\left(0.5d\theta_{ELV}\right),$$

(A13) $$u_{13:Triceps\;brachii\;lateral}^{max}=sig\left(0.5d\theta_{ELV}\right),$$

(A14) $$u_{14:Triceps\;brachii\;medial}^{max}=sig\left(0.5d\theta_{ELV}\right),$$

(A15) $$u_{15:Brachialis}^{max}=sig\left(-0.5d\theta_{ELV}\right),$$

(A16) $$u_{16:Brachioradialis}^{max}=sig\left(-0.5d\theta_{ELV}\right),$$

(A17) $$u_{17:Pronator\;teres}^{max}=sig\left(-0.2d\theta_{ELV}-0.5d\theta_{ELW}\right),$$

(A18) $$u_{18:Anconeus}^{max}=sig\left(0.2d\theta_{ELV}-0.2d\theta_{ELW}\right),$$

(A19) $$u_{19:Supinator}^{max}=sig\left(0.5d\theta_{ELW}\right),$$

(A20) $$u_{20:Pronator\;quadratus}^{max}=sig\left(-0.5d\theta_{ELW}\right).$$

The FCM–JA was described based on the angle differences of the five joint motions using the following Equations (A21)–(A49) for the right lower extremity.

(A21) $$u_{1:Psoas\;major}^{max}=sig\left(-0.5d\theta_{HPV}\right),$$

(A22) $$u_{2:Iliacus}^{max}=sig\left(-0.5d\theta_{HPV}\right),$$

(A23) $$u_{3:Gluteus\;maximus}^{max}=sig\left(0.5d\theta_{HPV}\right),$$

(A24) $$u_{4:Gluteus\;medius}^{max}=sig\left(-0.5d\theta_{HPU}\right),$$

(A25) $$u_{5:Gluteus\;minimus}^{max}=sig\left(-0.5d\theta_{HPU}\right),$$

(A26) $$u_{6:Inferior\;gemellus}^{max}=sig\left(-0.5d\theta_{HPW}\right),$$

(A27) $$u_{7:Obturator\;externus}^{max}=sig\left(-0.5d\theta_{HPW}\right),$$

(A28) $$u_{8:Obturator\;internus}^{max}=sig\left(-0.5d\theta_{HPW}\right),$$

(A29) $$u_{9:Superior\;gemellus}^{max}=sig\left(-0.5d\theta_{HPW}\right),$$

(A30) $$u_{10:Quadratus\;femoris}^{max}=sig\left(-0.5d\theta_{HPW}\right),$$

(A31) $$u_{11:Piriform}^{max}=sig\left(-0.5d\theta_{HPU}-0.5d\theta_{HPW}\right),$$

(A32) $$u_{12:Adductor\;brevis}^{max}=sig\left(-0.2d\theta_{HPV}+0.5d\theta_{HPU}-0.2d\theta_{HPW}\right),$$

(A33) $$u_{13:Adductor\;longus}^{max}=sig\left(-0.2d\theta_{HPV}+0.5d\theta_{HPU}-0.2d\theta_{HPW}\right),$$

(A34) $$u_{14:Adductor\;magnus}^{max}=sig\left(-0.2d\theta_{HPV}+0.5d\theta_{HPU}\right),$$

(A35) $$u_{15:Biceps\;femoris\;long\;head}^{max}=sig\left(0.5d\theta_{KNV}+0.5d\theta_{HPV}-0.5d\theta_{KNW}\right),$$

(A36) $$u_{16:Biceps\;femoris\;short\;head}^{max}=sig\left(0.5d\theta_{KNV}-0.5d\theta_{KNW}\right),$$

(A37) $$u_{17:Gracilis}^{max}=sig\left(0.2d\theta_{KNV}-0.2d\theta_{HPV}+0.5d\theta_{HPU}+0.2d\theta_{KNW}\right),$$

(A38) $$u_{18:Pectineus}^{max}=sig\left(-0.5d\theta_{HPV}+0.5d\theta_{HPU}\right),$$

(A39) $$u_{19:Rectus\;femoris}^{max}=sig\left(-0.5d\theta_{KNV}-0.5d\theta_{HPV}\right),$$

(A40) $$u_{20:Sartorius}^{max}=sig\left(0.2d\theta_{KNV}-0.2d\theta_{HPV}-0.2d\theta_{HPU}-0.2d\theta_{HPW}+0.2d\theta_{KNW}\right),$$

(A41) $$u_{21:Semimembranosus}^{max}=sig\left(0.5d\theta_{KNV}+0.5d\theta_{HPV}+0.2d\theta_{KNW}\right),$$

(A42) $$u_{22:Semitendinosus}^{max}=sig\left(0.5d\theta_{KNV}+0.5d\theta_{HPV}+0.2d\theta_{HPU}+0.5d\theta_{KNW}\right),$$

(A43) $$u_{23:Tensor\;fascia\;lata}^{max}=sig\left(-0.5d\theta_{HPV}-0.5d\theta_{HPU}+0.5d\theta_{HPW}-0.2d\theta_{KNW}\right),$$

(A44) $$u_{24:Vastus\;intermedius}^{max}=sig\left(-0.5d\theta_{KNV}\right),$$

(A45) $$u_{25:Vastus\;medialis}^{max}=sig\left(-0.5d\theta_{KNV}\right),$$

(A46) $$u_{26:Vastus\;lateralis}^{max}=sig\left(-0.5d\theta_{KNV}\right),$$

(A47) $$u_{27:Gastrocnemius}^{max}=sig\left(0.5d\theta_{KNV}\right),$$

(A48) $$u_{28:Plantaris}^{max}=sig\left(0.2d\theta_{KNV}\right),$$

(A49) $$u_{29:Popliteus}^{max}=sig\left(0.2d\theta_{KNV}\right).$$
